# Novel Animal Model of Enterobacteria Pathogenicity, Virulence, and Amoxicillin—Biosurfactant Synergic Using Nsombé (*Rhynchophorus phoenicis* Larvae)

**DOI:** 10.1002/mbo3.70025

**Published:** 2025-06-30

**Authors:** Sergy Patrick Junior Bissoko, Christian Aimé Kayath, Saturnin Nicaise Mokemiabeka, Frédéric Yannick Okouakoua, David Charles Roland Moukala, Duchel Jeanedvi Kinouani Kinavouidi

**Affiliations:** ^1^ Laboratoire de Biologie Cellulaire et Moléculaire (BCM), Faculté des Sciences et Techniques Université Marien Ngouabi Brazzaville Congo; ^2^ Institut National de Recherche en Sciences Exactes et Naturelles (IRSEN) Brazzaville Congo

**Keywords:** amoxicillin, biosurfactant, Enterobacteria, pathogenicity, *Rhynchophorus phoenicis*, synergy

## Abstract

Insect larvae are increasingly being employed as sophisticated infection models in the expanding field of pathogenic bacterial research. This innovative study aims to evaluate an alternative model for analyzing host‐pathogen interactions and assessing the efficacy of antimicrobial treatments using the biological system of *Rhynchophorus phoenicis* larvae. Using PCR techniques targeting 16S rRNA, virulence genes encoding Type III secretion system (T3SS) components, and the *Hsp60* gene, four major pathogenic Enterobacteriaceae were identified with 100% detection rates: *Salmonella Typhimurium*, *Klebsiella pneumoniae*, *Enterobacter cloacae*, and enteropathogenic *Escherichia coli* (EPEC). Virulence assessment revealed that injection of bacterial strains at a concentration of 10^5^ CFU/µL into *R. phoenicis* larvae was optimal for evaluating pathogenicity. Highly virulent strains—*E. coli* EPEC strain E2, *K. pneumoniae* K4, *S. Typhimurium* S4, and *E. cloacae* En2—caused significantly reduced larval survival, with bacterial loads in the hemolymph reaching up to 2.5 × 10^6^ CFU/µL. Treatment evaluation showed that the amoxicillin + biosurfactant combination was the most effective in prolonging larval survival across all time points. Survival rates peaked at 80% at 24 h and remained relatively high at up to 70% at 72 h for certain strains. In contrast, amoxicillin alone demonstrated variable and less sustained efficacy. These findings underscore the potential of *R. phoenicis* larvae as a valuable alternative model for exploring host–pathogen interactions and assessing the synergistic efficacy of combined antimicrobial treatments involving antibiotics and biosurfactants.

## Graphical abstract

This study highlights the potential of ***Rhynchophorus phoenicis*** larvae as an alternative infection model for evaluating host–pathogen interactions and the synergistic effects of combined antimicrobial therapies using antibiotics and biosurfactants.

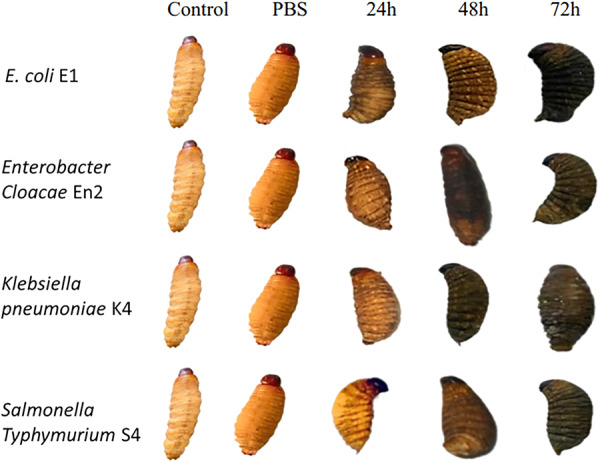

## 1 Introduction

Bacterial diarrheal diseases are a major public health problem, leading to increased morbidity and mortality in developing countries where illiteracy, poverty, overcrowding, poor sanitary conditions, and difficulties in accessing clean water are common (Ugboko et al. 2020).

Enterobacteria are a large family of Gram‐negative, rod‐shaped bacteria, comprising many species that are pathogenic to humans and animals, while others are intestinal commensals. Found in various environments, they play various roles that range from organic matter decomposition to host infection (Baldelli et al. 2021). Among pathogenic species, *Escherichia coli* is responsible for urinary tract infections, gastroenteritis, neonatal meningitis, and other infections (Whelan et al. 2023). *Salmonella* is known for causing salmonellosis as well as typhoid fever (Popa and Popa 2021). *Klebsiella* is involved in pneumonia, urinary tract infections, and sepsis (Abbas et al. 2024). Species of the *Enterobacter cloacae* complex are widely encountered in nature, but they can act as pathogens (Mezzatesta et al. 2012). The four species can use T3SS to invade and colonize mammalian (D'Aoust 1991; Hoffmann and Roggenkamp 2003; Dos Santos et al. 2020).

Type III secretion system (T3SS) is one of the nanomachine conserved among Gram‐negative bacteria and represents a crucial determinant of virulence for many pathogenic bacteria and some plant pathogens (Hueck 1998). This system is a biological pump that facilitates the translocation of virulence proteins from the bacterial cytoplasm directly into the host cell's cytosol through the plasma membrane (Schmidt and Hensel 2004).

Enterobacteria intensify virulence with antibiotic resistance. They have developed resistance mechanisms, including the production of extended‐spectrum beta‐lactamases (ESBLs), which hydrolyze a wide range of beta‐lactam antibiotics (Bahr et al. 2021). Enterobacteria have developed various mechanisms to resist amoxicillin, including the production of beta‐lactamases (De Angelis et al. 2020).

Combined antibiotic therapies delay the emergence of bacterial resistance and provide synergistic effects in the treatment of bacterial infections (Coates et al. 2020). The use of nanoparticles with antibiotics offers the advantage that if bacteria develop resistance to one component, the other component might eradicate them through a different mechanism (Hetta et al. 2023). Similarly, Biosurfactant, when administered with antibiotics, reduces bacterial survival and the likelihood of resistance development. This is achieved through the improved penetration of antibiotic molecules and a more rapid inhibitory effect (Ceresa et al. 2023).

To deepen the understanding of virulence mechanisms and develop new therapeutic strategies, it is crucial to have a suitable animal model that allows the examination of host‐pathogen interactions. In the early stages of research, it is especially important to choose a model that can be used in large numbers while minimizing ethical concerns. Due to the economic, technical, and ethical constraints associated with the extensive use of vertebrate animals, researchers are turning to invertebrate models such as *Drosophila melanogaster*, *Caenorhabditis elegans*, and *Galleria mellonella*, which are less subject to ethical restrictions (Wilson‐Sanders 2011; Kiani et al. 2022). These models possess an innate immune system similar to that of vertebrates, allowing for more accessible and cost‐effective studies of host‐pathogen interactions (Edwards and Kjellerup 2012). Among these models, *G. mellonella*, although less explored than others, is increasingly popular for studying bacterial virulence and the efficacy of antimicrobial treatments (Ménard 2021; Asai et al. 2023).

Palm weevil larvae (*R. phoenicis*) are large insects that belong to the Dryophthoridae family, the Rhynchophorinae subfamily, and the Rhynchophorini tribe (Wattanapongsiri 1965). These insects are holometabolous, meaning their biological cycle includes four distinct stages: egg, larva, pupa, and adult. The morphological characteristics and biological cycle of palm weevil have been extensively studied (Manee et al. 2023). Palm weevil larvae can be infected by pathogens primarily belonging to the families Bacillaceae, Pseudomonadaceae, Enterobacteriaceae, Streptococcaceae, and Micrococcaceae (Mazza et al. 2014).

In this study, we examined first of all *R. phoenicis* larvae as an animal model when infected by pathogens such as *Klebsiella* spp., *E. coli*, *Salmonella* spp. and *Enterobacter* spp. Secondly, we evaluate the effectiveness of the treatment that combined amoxicillin with a *Bacillus*‐derived biosurfactant against these Enterobacteriaceae in *R. phoenicis* larval model. The rationale for combining amoxicillin with a biosurfactant lies in enhancing antibacterial efficacy through improved membrane permeabilization, potential synergistic interactions, and a reduced likelihood of resistance development. Using the *R. phoenicis* larval model, we explored the potential benefits of combining amoxicillin with biosurfactants. This approach was guided by three main objectives: (1) To broaden the antibacterial spectrum—leveraging the complementary modes of action of antibiotics and biosurfactants to target a wider range of pathogens. (2) To help prevent the emergence of multidrug‐resistant strains—reducing the selective pressure exerted by antibiotics alone and thus limiting the development of resistance. (3) To enhance bactericidal efficacy through synergistic effects—improving bacterial killing rates by exploiting the membrane‐disrupting properties of biosurfactants in combination with the antimicrobial action of amoxicillin.

We hypothesize that *R. phoenicis* larvae can distinguish between highly virulent and less virulent Enterobacteriaceae strains and serve as a relevant model for evaluating the synergy between amoxicillin and a bacterially derived biosurfactant. To test this hypothesis, we assessed larval mortality and bacterial load following infection with different pathogenic strains, compared the efficacy of amoxicillin, the biosurfactant, and their combination on larval survival, and analyzed mortality reduction to detect a synergistic effect.

## 2 Materials and Methods

### 2.1 Acquisition of *R. Phoenicis* Larvae

The larvae of *R. phoenicis*, which are yellowish, apodous, measuring between 36 and 47 mm in length and 15–19 mm in width, and weighing approximately 4 g, were obtained from commercial suppliers. They were stored in darkness and used within 7 days of receipt.

### 2.2 Sample Collection

A total of 72 stool samples were obtained from three healthcare facilities: the COGEMO Medical Surgical Clinic, the TALANGAI Referral Hospital, and the Sino‐Congolese Hospital of Mfilou. Following collection, the samples were transported under refrigerated conditions (4°C) to the Department of Microbiology and Molecular Biology, where they underwent a series of microbiological and molecular analyses.

### 2.3 Strains and Culture Conditions

Using diarrheal stool samples, after a macroscopic examination to assess consistency and color, dilutions were prepared, and the resulting bacterial suspensions were streaked onto specific media: Hektoen enteric agar (HIMEDIA), EMB Levine agar (Liofilchem), and SS agar (HIMEDIA). Petri dishes were incubated at 37°C for 24 h. After initial isolation on the Petri dishes, different colonies were obtained. Each characteristic enterobacterial colony was isolated separately. The purification of the isolates was carried out rigorously through successive subcultures. Purity was assessed using a microscope for morphological characterization. The status was determined using 3% KOH. A genotype‐identified strain of *Bacillus safensis* was used for the production of Biosurfactant (Kaya‐Ongoto et al. 2019).

### 2.4 Pathogenicity Study of Enterobacterial Strains Isolated From Stool Samples

For this study, 20 enterobacterial strains, including 5 strains each of *E. coli*, *Enterobacter*, *Klebsiella*, and *Salmonella* isolated from diarrheal stool samples, were randomly selected. These strains were tested for their ability to emulsify hydrocarbons to determine their Biosurfactant production. The tests also evaluated biofilm formation, hemolysin production, swarming phenomenon, and resistance to acidity. These tests make it possible to characterize the pathogenic potential of each strain before injection into the host, thereby allowing prediction of their in vivo behavior.

### 2.5 Emulsification Index

The method adapted from Frédéric Yannick Okouakoua et al. (2024) was used with some adjustments. The turbidity of the bacterial cultures was standardized using McFarland standards. The emulsification index (EI24) was assessed by mixing the hydrocarbon with the acellular supernatant in a 1:1 (v/v) ratio. The mixture was homogenized using a vortex (model RSVA 10 Phoenix) at maximum speed for 10 min, then incubated for 24 h. The emulsification rate was determined by measuring the height of the emulsion layer. All experiments were carried out in triplicate.

### 2.6 Biofilm Formation Test

The ability of the isolates to form biofilms was assessed using Congo Red (CR) Agar and Crystal Violet (CV) assays, with modifications to the method developed by Frédéric Yannick Okouakoua et al. (2024). Biofilm formation was observed by the appearance of black colonies on CR Agar. The CV assay was performed on a 96‐well microtiter plate, following a previously established method (Borowicz et al. 2023). The results were recorded by calculating the average of three replicate measurements.

### 2.7 Hemolysin Production Test

To assess the ability of the isolates to produce hemolysins, a hemolysis test was performed. A total of 100 µL bacterial suspension was aseptically taken and inoculated into wells previously made on a blood agar plate containing 5% human blood. The plates were then incubated at 37°C for 24 h. Hemolytic activity was detected by the appearance of a halo around the wells, indicating the lysis of red blood cells.

### 2.8 Bacterial Swarming Test

The adapted method from Duchel Jeanedvi Kinouani Kinavouidi et al. (2022) was used with some modifications. Plates containing LB medium enriched with 0.5% glucose and 0.5% bacteriological agar were sterilized at 121°C for 15 min. After inoculating the enterobacteria strains, the plates were incubated at 30°C for 24 h. The plates were then examined to analyze the swarming phenomenon among the different strains.

### 2.9 Acidic pH Resistance Test

The method developed by Duchel Jeanedvi Kinouani Kinavouidi et al. was adapted with some modifications (Kinavouidi et al. 2022). The 24‐h bacterial culture was diluted 1:100 in LB medium acidified to pH 2 using HCl and then incubated at 37°C for 2 h. After incubation, a series of dilutions was performed, and 100 µL of each dilution was inoculated into EMB agar plates. The plates were incubated at 37°C for 24 h, and colony counting was used to assess the viability of the strains. The survival rate of bacterial cells grown at acidic pH was calculated using the following formula:

$$\%\;\mathrm{Survie}=\frac{\mathrm{Number}\;\mathrm{of}\;\mathrm{viable}\;\mathrm{cells}\;\mathrm{at}\;\mathrm{pH}2}{\mathrm{Number}\;\mathrm{of}\;\mathrm{viable}\;\mathrm{cells}\;\mathrm{at}\;\mathrm{neutral}\;\mathrm{pH}}\times 100.$$

### 2.10 Detection of Virulence Genes and Identification of Isolate

Matrix‐Assisted Laser Desorption Ionization Time of Flight (MALDI‐TOF) was used to identify a couple of *Enterobacteriaceae* isolates. DNA extraction and purification were performed using the NucleoSpin Microbial DNA kit (Macherey‐NAGEL). Bacterial isolates were cultured in 5 mL of LB broth for 24 h at 37°C with agitation. The quality of the extracted DNA was verified by agarose gel electrophoresis.

Amplification of the housekeeping 16S rRNA gene was performed by PCR (Thermal Cycler, Bio‐Rad) using the universal primers fD1 (5′‐AGACTTTGATCCTGGCTCAG‐3′) and rP2 (5′‐ACGGCTACCTTGTTACGACTT‐3′), yielding an amplicon of approximately 1500 base pairs, to further confirm bacterial identity.

Species identification for *Enterobacter* members was performed using hsp60 typing as previously described (Hoffmann and Roggenkamp 2003). Briefly, fragments of the hsp60 gene were amplified using primers Hsp60‐F (5′‐GGTAGAAGAAGGCGTGGTTGC‐3′) and Hsp60‐R (5′‐ATGCATTCGGTGGTGATCATCAG‐3′), generating an amplicon of approximately 264 base pairs, followed by sequencing of the PCR product.

For *Salmonella* and *E. coli* species, PCR and sequencing were used to amplify and sequence genes encoding the T3SS. Briefly, a reaction mix was prepared containing 2 ng/µL of DNA, 0.4 µM of each primer (Table 1), 200 µM of each dNTP, 1.25 units of OneTaq DNA polymerase, and 10 µL of OneTaq standard buffer (5X). Amplification was carried out in 30 cycles with an initial denaturation at 95°C for 30 s, followed by annealing at 55°C for 30 s and elongation at 72°C for 1 min 30 s, with a final extension at 72°C for 5 min. The resulting amplicons, ranging in size from 216 to 1782 bp (as detailed in Table 1), were separated by electrophoresis on a 1% agarose gel containing ethidium bromide, with Tris‐acetate‐EDTA (TAE) as the migration buffer. The results were visualized and photographed under UV light using a digital capture system. The PCR products were purified using the solution of Gel Extraction kit (Omega Bio‐tek), and the purified products were subjected to sequencing by the Sanger technique (3130 × l Genetic Analyser, Applied Biosystems). The sequences obtained were aligned with BioNumerics 7.5 (Applied Maths, Belgium) and manually corrected to resolve discrepancies between the sense and antisense strands. Sequences were compared with homologous sequences contained in public databases through NCBI using the BLASTn program. For each PCR assay, appropriate positive controls were included to ensure the reliability of the amplification reactions. For the 16S rRNA and hsp60 gene targets, well‐characterized reference strains from established collections were used as positive controls. In the case of T3SS genes—specifically SPI‐1, SPI‐2, and EPEC/EHEC‐associated genes—previously confirmed clinical isolates served as positive controls. Additionally, no‐template controls (NTCs) were systematically included in every PCR run to monitor for potential contamination of reagents or cross‐contamination during sample handling.

**Table 1:** List of primers used for PCR amplification of virulence genes in Salmonella enterica and *Escherichia coli* strains. Each primer pair targets a specific gene involved in the Type III Secretion System (T3SS).

Strains	Primers	Sequence (5′−3′)	Size (pb)	Target genes
*Salmonella* sp. SPI‐1	PrgI‐F	ATGTCGATTGCAACTATTGTCCCTG	306	PrgI
	PrgI‐R	TCATGAGCGTAATAGCGTTTCAACAG		
	SipD‐F	ATGCTTAATATTCAAAATTATTCCGC	1047	SipD
	SipD‐R	TTAATATCCTCTTCTGTTATCCTTGCAGG		
	SipB‐F	ATGGTAAATGACGCAAGTAGCATTAGCCG	1782	SipB
	SipB‐R	TTATGCGCGACTCTGGCGCAGAATAAA		
*Salmonella enterica* SPI‐2	SsaG‐F	ATGGATATTGCACAATTAGTGGATA	216	SsaG
	SsaG‐R	TCAGATTTTAGCAATGATTCCACTAA		
	SseB‐F	ATGTCTTCAGGAAACATCTTATGGG	591	SseB
	SseB‐R	TCATGAGTACGTTTTCTGCGCTATC		
	SseC‐F	ATGAATCGAATTCACAGTAATAGCG	1445	SseC
	SseC‐R	TTAAGCGCGATAGCCAGCTATTCTC		
EPEC/EHEC	EscF‐F	ATGAATTTATCTGAAATTACTCAAC	222	EscF
	EscF‐R	TTAAAAACTACGGTTAGAAATG		
	EspA‐F	ATGGATACATCAACTACAGCATCAG	579	EspA
	EspA‐R	TTATTTACCAAGGGATATTCCTG		
	EspD‐F	ATGCTTAATGTAAATAACGATATCC	1147	EspD
	EspD‐R	TTAAACTCGACCGCTGACAATACGG		

Abbreviations: bp, base pairs; EHEC, enterohemorrhagic *E. coli*; EPEC, enteropathogenic *E. coli*; SPI‐1/2, Salmonella Pathogenicity Island 1 and 2.

### 2.11 Evaluation of Enterobacteriaceae Virulence Using the *R. Phoenicis* Larvae Model

To evaluate the experimental infection model in *R. phoenicis* larvae, several parameters were examined: larval survival up to 72 h post‐inoculation, the development of *Enterobacteriaceae* strains in the hemolymph following infection, and the immune response assessed through larval melanization.

For each species, larvae were manually assigned to seven experimental groups: five groups corresponding to different tested bacterial strains, and two control groups. Each group contained 20 larvae, resulting in a total of 140 larvae per species.

Evaluations were performed every 24 h over a 3‐day period. The allocation of larvae to the groups was performed manually, without randomization. Additionally, assessments of survival and melanization were not conducted in a blinded manner, which represents a potential limitation of the study.

### 2.12 Determination of Bacterial Dose

The determination of the bacterial dose was carried out to identify the optimal concentration of each pathogenic strain required to induce a measurable and differentiated infectious response in *R. phoenicis* larvae. For this purpose, larval injections were performed with bacterial doses ranging from 10^4^ to 10^9^ CFU/larva. Twenty strains were tested (five strains each of *E. coli*, *Enterobacter*, *Klebsiella*, and *Salmonella*), each across six standardized dilutions.

For each dilution, 10 larvae were injected, amounting to 60 larvae per strain, which represents a total of 1200 larvae for the entire experiment. Each strain constituted a biological replicate, while the 10 larvae per condition served as technical replicates. This protocol allowed the identification of specific virulence profiles for each strain and the determination of the optimal bacterial concentration to be used in subsequent infection tests.

### 2.13 Preparation and Infection

Enterobacteriaceae strains were cultured on EMB Levine agar and subsequently transferred to Mueller‐Hinton broth until reaching an optical density of 0.5 at 600 nm. After centrifugation and washing, bacterial suspensions were adjusted to 10^5^ CFU per larva. The bacteria were injected into *R. phoenicis* larvae to simulate a controlled infection and evaluate the actual virulence of different Enterobacteriaceae strains in a living organism. Larvae were injected with 10 µL of each bacterial suspension directly into the hemolymph. Positive controls, negative controls, and PBS controls were included to validate the experiment. Larval feeding was halted to standardize experimental conditions. The larvae were then incubated at 37°C for 72 h in sterile glass jars, with daily monitoring to record mortality.

### 2.14 Quantification of Enterobacteriaceae in the Haematopoietic Hemolymph of Infected Larvae

This experiment was conducted to measure the ability of different strains to proliferate within the host, which is a critical indicator of virulence. It enables the distinction between highly virulent strains—capable of rapidly multiplying in the hemolymph—and those with moderate or low virulence. At specific time intervals (2, 4, 6, 12, and 24 h post‐infection), 20 µL of hemolymph were collected from each larva, serially diluted, and plated on nutrient agar. Colony‐forming units (CFUs) were counted to quantify the bacterial load in the hemolymph. This time‐course monitoring of bacterial burden directly tests the hypothesis that *Rhynchophorus phoenicis* larvae can discriminate between enterobacterial strains based on virulence level, by exhibiting differentiated infection responses. The resulting data also provide a baseline for evaluating the effectiveness of antimicrobial treatments, particularly the combination of amoxicillin and biosurfactant.

### 2.15 Evaluation of Melanization

The evaluation of melanization was conducted to assess the innate immune response of *R. phoenicis* larvae following bacterial infection. Melanization is a key component of the insect's defense system, associated with pathogen recognition and encapsulation. The presence and intensity of melanization provide an indirect but reliable indicator of the activation of the immune system in response to infection. This observation was carried out macroscopically and documented through photographs, using five larvae per experimental condition. By comparing melanization patterns induced by different bacterial strains, this assay helped to distinguish highly virulent strains—eliciting stronger immune responses—from less virulent ones. Furthermore, it contributed to testing our hypothesis by demonstrating whether the larvae could mount differential immune responses to high‐ versus low‐virulence strains, thereby supporting their ability to discriminate between them.

### 2.16 Biosurfactant Production Assay for *Bacillus* Strain

This assay was performed to determine whether the *Bacillus* strain used in this study was capable of producing biosurfactants with emulsifying and antibacterial properties. This step was essential to test our hypothesis that combining amoxicillin with a bacterial biosurfactant could produce a synergistic effect, enhancing larval survival following infection with pathogenic Enterobacteriaceae strains. Biosurfactant extraction followed the protocol described by Kinouani Kinavouidi et al. (2022), and the product was evaluated for its emulsification ability—a marker of biosurfactant presence—and its antibacterial activity. The biosurfactant extract was tested against eight reference pathogenic strains (*Klebsiella*, *P. aeruginosa*, *S. aureus*, *E. coli*, *S. typhimurium*, *S. flexneri* 5a M90T, *B. cereus*, and *Enterobacter*), according to the method of Bokamba Moukala et al (Okouakoua et al. 2024). Demonstrating antimicrobial activity supported the role of the biosurfactant as an active component in the combined treatment. This experiment, therefore, contributes to validating the potential of biosurfactant‐amoxicillin synergy in the larval infection model and helps elucidate the mechanism of action.

### 2.17 Combined Effect of *Bacillus* Biosurfactant and Amoxicillin Against Enterobacteria

The combined effect of *Bacillus*‐derived biosurfactant and amoxicillin was investigated to evaluate whether their association enhances antibacterial activity against enterobacterial strains. Preparing a stock solution of amoxicillin at a precise concentration (10 mg/mL) and determining the minimum inhibitory concentration (MIC) of amoxicillin alongside the effective threshold of the biosurfactant allowed for controlled, reproducible testing conditions. By standardizing the bacterial suspension's absorbance at 600 nm, we ensured consistent bacterial load across experiments. This setup tests the hypothesis that the biosurfactant and amoxicillin act synergistically to improve treatment efficacy against pathogenic Enterobacteriaceae, as reflected in increased bacterial inhibition and improved larval survival in subsequent infection assays.

### 2.18 Efficacy Threshold of Biosurfactant

To evaluate the antimicrobial efficacy of the *Bacillus*‐derived biosurfactant, tubes containing 2 mL of LB medium were prepared with increasing biosurfactant volumes (20–100 µL, in 20 µL increments). Each tube was inoculated with 100 µL of bacterial suspension from the tested pathogens and incubated at 37°C for 24 h. After incubation, 100 µL from each tube was plated onto LB agar. CFUs were counted after overnight incubation to assess bacterial viability. The lowest biosurfactant volume at which no colonies appeared was recorded as the efficacy threshold, indicating complete growth inhibition.

### 2.19 MIC of Individual Amoxicillin

The determination of the MIC of amoxicillin was conducted to establish the lowest concentration capable of inhibiting the growth of each bacterial strain. The MIC of amoxicillin was determined using the Mueller‐Hinton broth microdilution method, with a concentration range of 5–2000 µg/mL. The MIC was considered the lowest concentration of amoxicillin at which no bacterial colonies were observed on the plate. Sub‐lethal concentrations of both bioactive compounds (amoxicillin and Biosurfactant) were identified based on these results and used to evaluate their combined effect.

### 2.20 Evaluation of Combined Action of Amoxicillin and Biosurfactant

The evaluation of the combined action of amoxicillin and biosurfactant was performed to test the hypothesis that their combination produces a synergistic effect, enhancing antimicrobial efficacy against enterobacterial strains in *R. phoenicis* larvae. By comparing the effects of amoxicillin alone, biosurfactant alone, and their combination on larval survival and bacterial load, we aimed to determine whether the combined treatment improves larval survival more effectively than individual treatments. This experiment directly addresses the hypothesis by assessing whether the synergy between amoxicillin and biosurfactant can reduce bacterial virulence and increase host survival.

The combined effect of Bacillus Biosurfactant and amoxicillin was evaluated using the modified method developed by Kasturi Joshi‐Navare and Asmita Prabhune (Joshi‐Navare and Prabhune 2013a). In summary, four tests were conducted. Biosurfactant and amoxicillin extracts were carefully mixed with sterile distilled water and a bacterial suspension to expose the cells to the combined action of both agents. The mixtures were incubated at 37°C with shaking at 180 rpm for 24 h. The samples were taken at 2, 4, and 6 h, and CFUs were determined by plating 50 µL of each mixture on nutrient agar plates (Table 2). After incubation at 37°C for 24 h, the colonies were counted. All tests were performed in triplicate to ensure result reproducibility. A control was included with cells that were not exposed to any bioactive agents. The dilution scheme is detailed in the table below.

**Table 2:** Reaction mixtures used for biosurfactant efficacy testing. Each condition combines a bacterial suspension with amoxicillin (250 µg/mL), biosurfactant, or both, and is brought to a final volume of 2000 µL with sterile distilled water.

Number of reactions	Reactions	Volume (μL)				
		Bacterial suspension	Amoxicillin 250 μg/mL	Biosurfactant	Sterile distilled water	Total
1	Control	100	/	/	1900	2000
2	Amoxicillin	100	100 µL	/	1800	2000
3	Biosurfactant	100	/	40 µL	1860	2000
4	Amoxicillin + Biosurfactant	100	100 µL	40 µL	1760	2000

Abbreviations: AMX, amoxicillin; BS, biosurfactant; µL, microliters.

### 2.21 Survival Test of *R. Phoenicis* Larvae in the Presence of Amoxicillin and Biosurfactant

The survival test of *R. phoenicis* larvae in the presence of amoxicillin and biosurfactant was conducted to evaluate the effectiveness of these treatments—alone and in combination—on larval survival following infection by pathogenic Enterobacteriaceae strains. This experiment tests the hypothesis that the larvae can reveal a synergistic effect between amoxicillin and the biosurfactant by comparing survival rates across treatment groups. By monitoring larval survival over time, we can assess whether the combined treatment improves larval resistance to infection more than each treatment alone, thereby validating the model's ability to discriminate treatment efficacy and confirm synergistic antimicrobial activity.

*R. phoenicis* larvae used for the survival test with amoxicillin and biosurfactant were first externally disinfected with 70% ethanol. The larvae were then randomly divided into four groups: larvae infected only with the bacterial strain, larvae infected with the strain and amoxicillin, larvae infected with the strain and biosurfactant, and larvae infected with the strain, amoxicillin, and biosurfactant.

For infection, 10 µL (approximately 10^5^ CFU/mL) of each mixture was injected into the hemolymph using an insulin syringe. Infected larvae were placed in sterile containers and incubated at 37°C. Mortality was monitored daily for 3 days. Ten larvae were used for each experimental condition, with controls including non‐injected larvae and larvae injected with 10 µL of sterile PBS.

### 2.22 Statistical Analysis

GraphPad Prism 8 and Excel software were used for the analysis. Data represent the arithmetic means of at least three repetitions. Data were expressed as mean ± standard deviation, and the Kruskal–Wallis test was used to determine statistical differences, with *p* < 0.05 considered significant.

## 3 Results

### 3.1 Profiling of Stool Samples Bacterial Isolation

The macroscopic characteristics of the stool samples, including color and consistency, were carefully examined. The analysis revealed that the majority of samples exhibited a mucous or liquid consistency, typically with a yellowish hue. Brown stools were the most frequently observed, followed by yellowish stools, whereas greyish, green, and black stools were considerably less common. These findings underscore notable variability in gastrointestinal status among patients, with a high prevalence of abnormal stool consistency and coloration, indicative of conditions such as diarrhea (data not shown).

The distribution of stool samples by collection site showed some heterogeneity: 20 samples (27.78%) were obtained from the Hôpital de Référence de Talanga, 28 samples (38.89%) from the Clinique Médico‐Chirurgicale COGEMO, and 24 samples (33.33%) from the Hôpital Sino‐Congolais de Mfilou.

Microbiological analysis indicated that *Salmonella* spp. were the predominant bacterial genus, isolated in 45.83% of the samples, followed by *E. coli* (22.22%), *Klebsiella* spp. (19.44%), and *Enterobacter* spp. (12.50%). This distribution highlights *Salmonella* as the primary etiological agent among the isolates, with *E. coli*, *Klebsiella*, and *Enterobacter* also contributing significantly to the observed infections.

### 3.2 Analysis of the Pathogenicity of Isolates

#### 3.2.1 Screening of Virulence Genes

In this study, bacterial strains isolated from stool samples were identified using MALDI‐TOF mass spectrometry and 16S rRNA gene sequencing (amplicon size ~1500 bp, primers fD1/rP2) for *K. pneumoniae*. *E. cloacae* identification was confirmed by amplification of the ***hsp60*** gene (264 bp, primers Hsp60‐F/Hsp60‐R). *S. Typhimurium* and enteropathogenic *E. coli* (EPEC) were identified by targeting genes encoding components of the T3SS.

Virulence gene profiling revealed distinct patterns across *E. coli* (E1–E5) and *Salmonella* (S1–S5) isolates. All *E. coli* strains harbored the **EscF** gene (222 bp), whereas *EspA* (579 bp) and EspD (1147 bp) were not detected. Among *Salmonella* strains, PrgI (306 bp), SipD (1047 bp), and SsaG (216 bp) were consistently absent. The SipB gene (1782 bp) was detected in strains S1, S4, and S5, but not in S2 or S3. The SseB gene (591 bp) was present in all strains except S5, while SseC (1445 bp) was found in S1, S2, and S3 only (Table 3).

**Table 3:** Virulence gene profiles of *E. coli* (E1–E5) and Salmonella (S1–S5) strains, based on PCR detection.

Strains	Virulence gene profile								
	EscF	EspA	EspD	IPS‐1			IPS‐2		
				PrgI	SipD	SipB	SsaG	SseB	SseC
E1	+	−	−	NA	NA	NA	NA	NA	NA
E2	+	−	−	NA	NA	NA	NA	NA	NA
E3	+	−	−	NA	NA	NA	NA	NA	NA
E4	+	−	−	NA	NA	NA	NA	NA	NA
E5	+	−	−	NA	NA	NA	NA	NA	NA
S1	NA	NA	NA	−	−	+	−	+	+
S2	NA	NA	NA	−	−	−	−	+	+
S3	NA	NA	NA	−	−	−	−	+	+
S4	NA	NA	NA	−	−	+	−	+	−
S5	NA	NA	NA	−	−	+	−	−	−

*Note:* +: gene detected (present), −: gene not detected, NA: not applicable (gene not relevant for this species). EscF, EspA, EspD: genes associated with the Type III secretion system (T3SS) in *E. coli* (Enteropathogenic *E. coli*, EPEC). PrgI, SipD, SipB, SsaG, SseB, SseC: genes associated with Salmonella Pathogenicity Islands 1 and 2 (SPI‐1 and SPI‐2) in *S. enterica*.

The colors represent the following features: Sky: represents the names of the isolates, Cream: represents the genes that are integral components of the type III secretion system of *E. coli*, Pistachio: represents the genes from the first pathogenicity island (IPS‐1) of the type III secretion system of S. enterica, Peach: represents the genes from the second pathogenicity island (IPS‐2) of the type III secretion system of S. enterica.

Sequencing of these virulence markers confirmed species‐level identification with 100% identity: *E. coli* strain E2 was classified as EPEC, *Salmonella* strain S4 as *S. Typhimurium*, *Klebsiella* strain K4 as *K. pneumoniae*, and *Enterobacter* strain En2 as *E. cloacae*.

The absence of certain T3SS genes in *Salmonella* isolates (e.g., PrgI, SipD, SsaG) may reflect underlying genetic variability among clinical strains, possibly due to partial deletions or divergence within *Salmonella* Pathogenicity Islands (SPI‐1 and SPI‐2). Alternatively, technical factors such as suboptimal PCR conditions, primer mismatch, or low‐quality DNA may have contributed to the non‐detection of these genes. However, the successful amplification of other T3SS genes (SipB, SseB, SseC) in the same isolates supports the hypothesis of strain‐specific variation in virulence gene content rather than systematic methodological failure (Table 3).

#### 3.2.2 Biosurfactant Production

**Figure 1:**
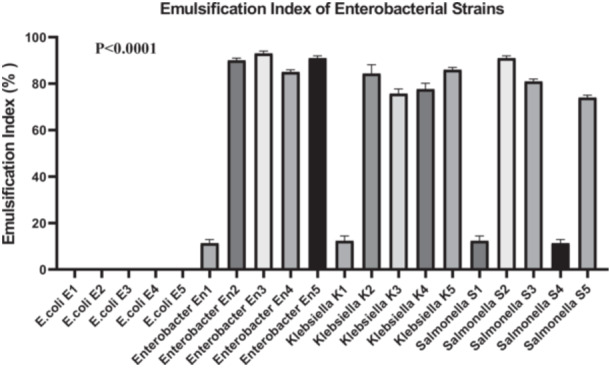
Emulsification index of enterobacterial strains. The y‐axis expresses the percentage of the presence of biosurfactant or not, while the x‐axis shows the different isolates used in this study including *E. coli* strains (E1, E2, E3, E4, and E5), Enterobacter strains (En1, En2, En3, En4, and En5), Klebsiella strains (K1, K2, K3, K4, and K5), Salmonella strains (S1, S2, S3, S4, and S5). Values represent mean ± SD (*n* = 3). EI_42_ reflects biosurfactant production capacity. A significant difference was observed between groups (*p* < 0.0001). EI_42_ = Emulsification Index at 24 h; % = Percentage.

To assess biosurfactant production, the emulsification index at 24 h (EI_42_) was measured using the cell‐free supernatants of each enterobacterial strain. All strains, except those of *E. coli*, demonstrated the ability to emulsify hydrocarbons, with EI_42_ values ranging from 10% to 100% (Figure 1). *E. coli* strains exhibited no emulsifying activity (0 ± 0), whereas *Enterobacter* and *Klebsiella* strains showed significantly higher indices, such as En2 (90 ± 1), En3 (93 ± 1), and *Klebsiella* K5 (86 ± 1). *Salmonella* strains displayed intermediate emulsification indices, ranging from 12.33 ± 2.08 to 91 ± 1.

A one‐way ANOVA was performed to compare EI_42_ values across all 20 bacterial strains. The analysis revealed a highly significant difference among groups (*F*_(19, 40)_ = 2165, *p* < 0.0001), indicating that emulsification capacity is strongly strain‐dependent. Between‐group variability accounted for the vast majority of the total variance (SS = 95,313 vs. residual SS = 92.67). Homogeneity of variances was confirmed using Bartlett's test (*p* > 0.05), suggesting no significant violation of ANOVA assumptions. A post hoc Dunn's test with Bonferroni correction was applied to identify specific intergroup differences. The results confirmed that *Enterobacter* and *Klebsiella* strains had significantly higher EI_42_ values compared to *E. coli* strains (*p* < 0.0001), supporting their superior biosurfactant production potential.

#### 3.2.3 Overview of Biofilm Formation by All Isolates

The ability of enterobacterial strains to form biofilms was evaluated using both CR agar and CV staining assays. All isolates (100%, 20/20) tested positive for biofilm production on CR agar.

Based on quantitative CV staining, 65% of strains (13/20) exhibited strong biofilm formation (+++), 25% (5/20) moderate (++), and 10% (2/20) weak biofilm production (+). These results underscore species‐dependent differences in biofilm‐forming capacity, which may contribute to their differential pathogenicity and antibiotic tolerance (Table 4).

**Table 4:** Biofilm formation capacity of enterobacterial strains evaluated using crystal violet (CV) staining and Congo Red (CR) agar.

Strain	Crystal violet assay	Congo red agar
*E. coli* E1	++	+
*E. coli* E2	+	+
*E. coli* E3	+++	+
*E. coli* E4	++	+
*E. coli* E5	+++	+
*Enterobacter* En1	+++	+
*Enterobacter* En2	+++	+
*Enterobacter* En3	+++	+
*Enterobacter* En4	+	+
*Enterobacter* En5	+++	+
*Klebsiella* K1	+++	+
*Klebsiella* K2	++	+
*Klebsiella* K3	+++	+
*Klebsiella* K4	+++	+
*Klebsiella* K5	+++	+
*Salmonella* S1	++	+
*Salmonella* S2	++	+
*Salmonella* S3	+++	+
*Salmonella* S4	+++	+
*Salmonella* S5	+++	+

*Note:* (+++): Strong biofilm formation, (++): Moderate biofilm formation, (+): Weak biofilm formation.

#### 3.2.4 Bacterial Swarming Capacity of Enterobacterial Strains

To evaluate the swarming motility of enterobacterial strains isolated from diarrheic stool samples, a swarming assay was performed. The results indicated that all tested strains exhibited swarming behavior on semi‐solid agar (data not shown).

#### 3.2.5 Hemolysis Test

**Figure 2:**
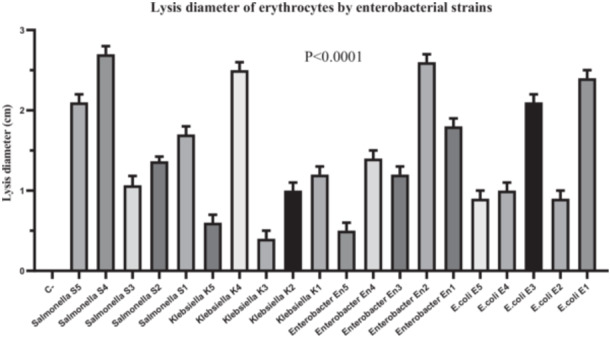
Analysis of the pathogenicity of isolates using the hemolysis test. The y‐axis represents the diameters in centimeter, while the x‐axis shows the different isolates used in this study including *E. coli* (strain E1, E2, E3, E4, and E5), Enterobacter (strain En1, En2, En3, En4, and En5), Klebsiella (strain K1, K2, K3, K4, and K5), Salmonella (S1, S2, S3, S4, and S5). Hemolytic zone diameters are expressed as mean ± standard deviation (SD). A significant difference was observed between groups (*p* < 0.0001). cm = centimeter, SD = standard deviation.

To evaluate the hemolytic activity of the bacterial strains, the diameter of the lysis zone on blood agar was measured. The results showed distinct variations among species. *E. coli* strains exhibited low hemolytic activity, with mean diameters of 2.4 ± 0.1 mm for E1, 0.9 ± 0.1 mm for E2, 2.1 ± 0.1 mm for E3, 1.0 ± 0.1 mm for E4, and 0.9 ± 0.1 mm for E5. In contrast, *Enterobacter* and *Klebsiella* strains showed significantly greater hemolytic activity, particularly En2 (2.6 ± 0.1 mm) and K4 (2.5 ± 0.1 mm). *Salmonella* strains displayed intermediate values, ranging from 1.7 ± 0.1 mm (S2) to 2.7 ± 0.1 mm (S4) (Figure 2).

A two‐way ANOVA was performed to assess the effect of strain identity (row factor) and species (column factor) on hemolytic activity. The overall model revealed that both factors contributed significantly to the variance in hemolytic activity (strain: *F*_(2, 40)_ = 8.32, *p* = 0.0010; species: *F*_(20, 40)_ = 259.03, *p* < 0.0001). Multiple comparisons were then conducted using Tukey's post hoc test, which confirmed significantly higher hemolytic activity in strains En2, K4, and S4 compared to *E. coli* strains E2, E4, and E5 (*p* < 0.01) (Figure 2).

#### 3.2.6 Acid Resistance Test

**Figure 3:**
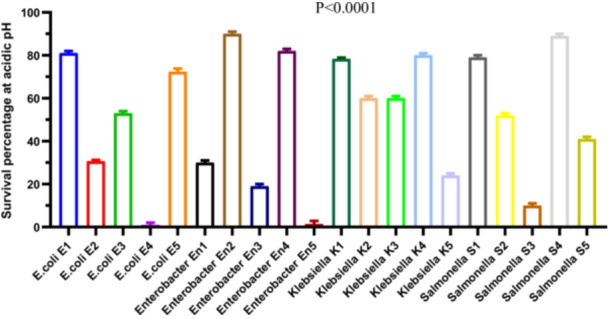
Survival percentage at pH = 2. The y‐axis represents the survival percentage, while the x‐axis shows the different isolates used in this study including *E. coli* (strain E1, E2, E3, E4, and E5), Enterobacter (strain En1, En2, En3, En4, and En5), Klebsiella (strain K1, K2, K3, K4, and K5), Salmonella (S1, S2, S3, S4, and S5). Each bar represents the mean ± standard deviation (SD) of three replicates (*n* = 3). A significant difference was observed between strains (*p* < 0.0001). pH = potential of hydrogen, SD = standard deviation.

To evaluate acid resistance, 20 bacterial strains were exposed to acidic conditions (pH 3.0), and survival rates were measured. A total of 18 out of 20 strains (90%) demonstrated the ability to survive under acidic stress (Figure 3). All *Klebsiella* and *Salmonella* strains exhibited complete survival (100%). In contrast, *E. coli* strains showed variable survival rates ranging from 1.0 ± 1.0% to 72.3 ± 0.88%, while *Enterobacter* strains ranged from 19.0 ± 1.0% to 90.0 ± 1.0% (Figure 3).

A repeated‐measures ANOVA (Geisser‐Greenhouse correction, ε = 0.09373) indicated a highly significant effect of bacterial group on acid resistance (*F*_(1.781, 3.562)_ = 2439, *p* < 0.0001, *R* ^2^ = 0.9992). However, no significant effect was attributed to individual matching (*F*_(2, 38)_ = 0.195, *p* = 0.8237), indicating consistency across replicates. Post hoc comparisons using Dunn's multiple comparison test confirmed that survival rates of *Klebsiella* and *Salmonella* strains were significantly higher (*p* < 0.01) than those of *E. coli* and *Enterobacter* strains. These findings demonstrate statistically robust differences in acid tolerance among the tested genera, with *Klebsiella* and *Salmonella* strains showing superior acid resistance under the tested conditions (Figure 3).

**Figure 4:**
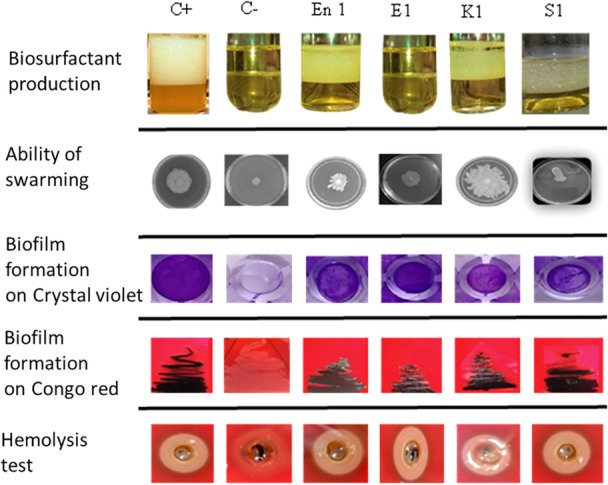
Biological characteristics of different strains of enterobacteria. C+: Positive control, C−: Negative control, En1: Enterobacter strain En1, E1: *E. coli* strain E1, K1: Klebsiella K1 and S1: Salmonella S1. This figure highlights biosurfactant production, bacterial swarming ability, biofilm formation in the presence of crystal violet and Congo red, and hemolysis activity on blood agar.

To assess the pathogenicity of the strains, we conducted five experiments, including tests for biosurfactant production, bacterial swarming ability, biofilm formation in the presence of CV and CR, and hemolytic activity on blood agar (Figure 4). The biological profiles of the tested strains highlight their respective properties. A positive control (C^+^) and a negative control (C^−^) were included to validate the experimental conditions (Figure 4). The analyzed strains include *Enterobacter* (En1), *E. coli* (E1), *Klebsiella* (K1), and *Salmonella* (S1). These results provide a comparative overview of the biological behavior of each strain under controlled conditions (Figure 4).

### 3.3 Investigation of *R. Phoenicis* Larvae as an Animal Model

#### 3.3.1 Impact of Bacterial Dose on Mortality of *R. Phoenicis* Larvae

One of the objectives of this study was to evaluate the virulence of Enterobacteriaceae strains isolated from diarrhoeal stool samples in *R. phoenicis* larvae and to determine the optimal dose for virulence experiments using this insect model.

The results showed that at concentrations of 10^7^ CFU/larvae or higher, all larvae died within 24 h. In contrast, at a concentration of 10^4^ CFU/larvae, mortality was either zero or nearly zero. Concentrations of 10^5^ and 10^6^ CFU/larvae induced an intermediate level of virulence, allowing differentiation of the virulence potentials of different strains. The dosage of 10^5^ CFU/larvae was selected for the tests in this study.

#### 3.3.2 Characterization of Virulence of Enterobacteriaceae Strains in *R. Phoenicis* Larvae

##### 3.3.2.1 E. Coli

**Figure 5:**
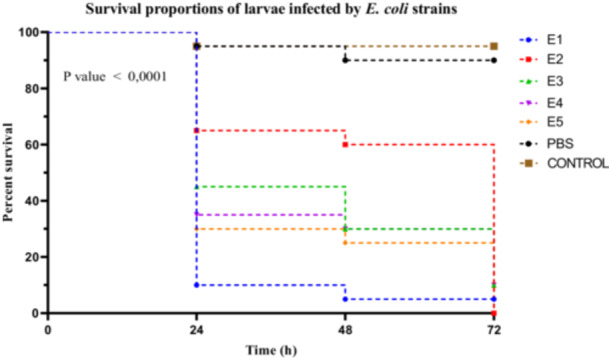
Kaplan–Meier survival curves of *R. phoenicis* larvae infected with *E. coli* strains (E1, E2, E3, E4, and E5) over 72 h post‐infection. Survival percentages are indicated at 24, 48, and 72 h. A significant difference in survival was observed between groups (*p* < 0.0001). h = hours post‐infection.

*E. coli* strains exhibited marked variability in virulence, as demonstrated by the Kaplan–Meier survival curves (Figure 5). Strain E1 was highly virulent, inducing rapid larval mortality with a survival rate of only 10% at 24 h. Strains E3, E4, and E5 were classified as moderately virulent, with respective survival rates of 45%, 35%, and 30%, while strain E2, with a survival rate of 65%, was considered weakly virulent.

Statistical analysis using the log‐rank (Mantel–Cox) test revealed a highly significant difference among the survival curves of the different strains (*χ* ^2^ = 94.53, df = 6, *p* < 0.0001). This heterogeneity was further supported by the log‐rank test for trend (*χ* ^2^ = 62.13, df = 1, *p* < 0.0001), indicating a significant trend in virulence across the strains. Additionally, the Gehan–Breslow–Wilcoxon test reinforced these findings (*χ* ^2^ = 80.48, df = 6, *p* < 0.0001), suggesting that the differences were particularly pronounced in the early stages of infection. A post hoc Dunn's test, applied following a Kruskal–Wallis test (see Section 3.3.3), enabled the differentiation of strain groups based on their virulence levels, statistically confirming the classification of E1 as highly virulent, E3 to E5 as moderately virulent, and E2 as weakly virulent (Figure 5).

##### 3.3.2.2 Enterobacter Sp

**Figure 6:**
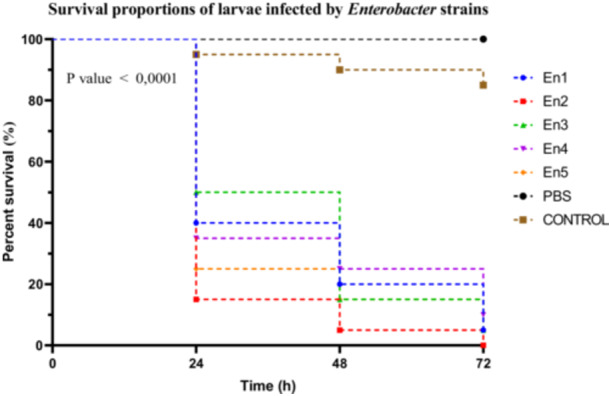
Kaplan–Meier survival curves of *R. phoenicis* larvae infected with Enterobacter strains (En1, En2, En3, En4, and En5) over 72 h post‐infection. Survival percentages are shown at 24, 48, and 72 h. A significant difference in survival was observed between groups (*p* < 0.0001). h = hours post‐infection.

Among the *Enterobacter* spp. strains, significant differences in virulence were observed between groups. Strain En2 induced marked larval mortality, with a survival rate of 15% at 24 h, classifying it as highly virulent. Strains En1, En4, and En5 were considered moderately virulent, with survival rates of 40%, 35%, and 25%, respectively. In contrast, strain En3 showed low virulence, with a larval survival rate of 50% (Figure 6).

Larval survival curves following infection with the different strains were analyzed using the Kaplan–Meier method, and comparisons revealed statistically significant differences between groups (Mantel–Cox log‐rank test: *χ* ^2^ = 100.3, df = 6, *p* < 0.0001). This trend was further supported by the log‐rank test for trend (*χ* ^2^ = 64.76, df = 1, *p* < 0.0001), indicating a significant progression of virulence within the genus. The Gehan–Breslow–Wilcoxon test also showed significant differences among survival curves (*χ* ^2^ = 83.82, df = 6, *p* < 0.0001), highlighting the differential impact of the strains on larval mortality. A post hoc Dunn's test, corrected for multiple comparisons, identified strains En2 and En5 as significantly more virulent than En3 (*p* < 0.05).

##### 3.3.2.3 Salmonella Sp

**Figure 7:**
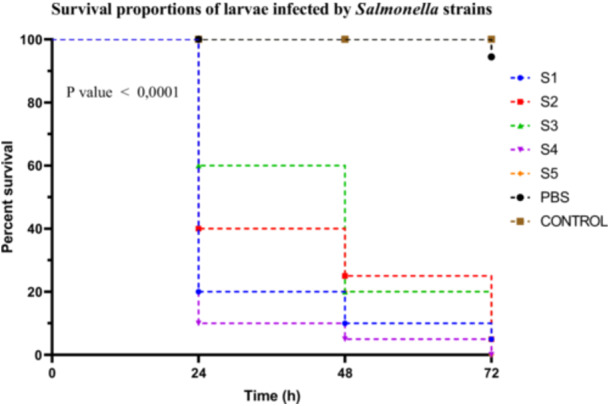
Kaplan–Meier survival curves of *R. phoenicis* larvae infected with Salmonella strains (S1, S2, S3, S4, and S5) over 72 h post‐infection. Survival percentages are shown at 24, 48, and 72 h. A significant difference in survival was observed between groups (*p* < 0.0001). h = hours post‐infection.

*Salmonella* spp. strains exhibited distinct virulence profiles based on Kaplan–Meier survival curves assessed over 72 h. Strain S4 was classified as highly virulent, causing a larval survival rate of only 10%. Strains S1, S2, and S5 were moderately virulent, with survival rates of 20%, 40%, and 40%, respectively. In contrast, strain S3 demonstrated low virulence, with a survival rate of 60%. Survival curve analysis using the log‐rank (Mantel–Cox) test revealed a highly significant difference between groups (*χ* ^2^ = 113.8, df = 6, *p* < 0.0001). This result was confirmed by the log‐rank test for trend (*χ* ^2^ = 70.84, df = 1, *p* < 0.0001), indicating a progressive decrease in survival corresponding to the increasing virulence of the strains. The Gehan–Breslow–Wilcoxon test also supported these findings (*χ* ^2^ = 94.19, df = 6, *p* < 0.0001), highlighting the robustness of the observed differences, especially in the early stages post‐infection (Figure 7). No post hoc correction was applied in this case, as multiple comparisons were addressed by stratifying the strains into virulence groups based on predefined survival percentage thresholds.

##### 3.3.2.4 Klebsiella Sp

**Figure 8:**
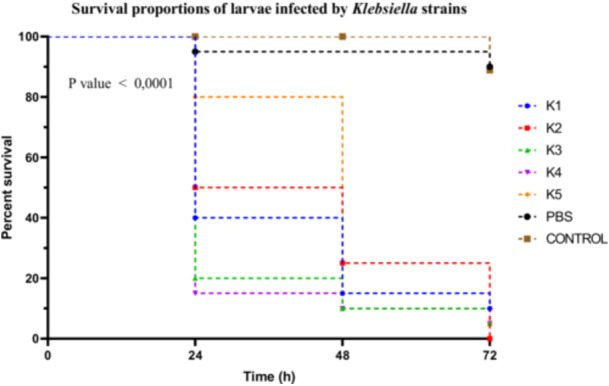
Kaplan–Meier survival curves of *R. phoenicis* larvae infected with Klebsiella strains (K1, K2, K3, K4, and K5) over 72 h post‐infection. Survival percentages are shown at 24, 48, and 72 h. A significant difference in survival was observed between groups (*p* < 0.0001). h = hours post‐infection.

*Klebsiella* spp. strains exhibited distinct virulence profiles, as shown by the Kaplan–Meier survival curves. Strain K4 was the most virulent, inducing a larval survival rate of 15% at 24 h. Strains K1, K2, and K3 were classified as moderately virulent, with survival rates of 40%, 50%, and 20%, respectively. In contrast, strain K5 showed very low virulence, with an 80% survival rate (Figure 8).

Statistical analysis of the survival curves using the log‐rank (Mantel–Cox) test revealed significant differences among the strains (*χ* ^2^ = 97.18; df = 6; *p* < 0.0001). The log‐rank test for trend also indicated a significant trend in virulence variation (*χ* ^2^ = 60.99; df = 1; *p* < 0.0001). These results were confirmed by the Gehan–Breslow–Wilcoxon test (*χ* ^2^ = 85.63; df = 6; *p* < 0.0001). A Dunn's post hoc correction was applied following the Kruskal–Wallis test for multiple comparisons among strains, showing that the differences between K4 and K5, as well as between K3 and K2, were statistically significant (*p* < 0.01).

#### 3.3.3 Evaluation of Bacterial Load of Enterobacteriaceae in the Hemolymph of Larvae

The ability of the different strains to infect, persist, and multiply in *R. phoenicis* larvae was assessed by counting CFUs per 10 µL of hemolymph at various time points post‐infection. The appearance of the hemolymph from infected larvae was compared to that of non‐infected larvae. The tube containing hemolymph from an infected larva showed a darker liquid, whereas that from a non‐infected larva appeared yellow (data not shown).

##### 3.3.3.1 E. Coli

**Figure 9:**
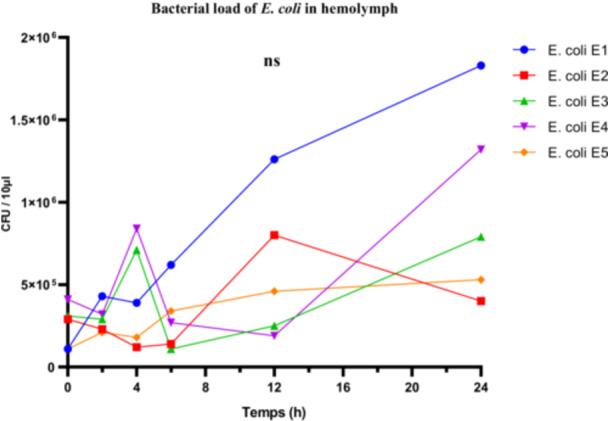
Bacterial load (CFU/10 µL hemolymph) in *R. phoenicis* larvae following infection with *E. coli* strains (E1, E2, E3, E4, and E5). Statistical analysis showed no significant difference between groups (ns). CFU = colony‐forming units, ns = non‐significant.

*E. coli* strains exhibited variable proliferation capacities in the hemolymph of *R. phoenicis* larvae. Strain E1, classified as highly virulent based on larval survival rate, reached a maximum bacterial load of 1.8 × 10^5^ CFU/µL. The moderately virulent strains E3 and E4 reached 0.7 × 10^5^ and 1.3 × 10^5^ CFU/µL, respectively, while the low‐virulence strain E2 did not exceed 0.4 × 10^5^ CFU/µL (Figure 9).

Despite these apparent differences, one‐way ANOVA did not reveal any statistically significant differences in the mean bacterial loads across the five strains (*F*_(4, 25)_ = 1.455; *p* = 0.2457; *R* ^2^ = 0.1888), suggesting that the observed variability may be due to intra‐group fluctuations. The Brown‐Forsythe test confirmed no significant difference in variance homogeneity (*F*_(4, 25)_ = 1.178; *p* = 0.3446), while Bartlett's test revealed significant heterogeneity in variances (corrected statistic = 9.884; *p* = 0.0424), calling for caution in interpreting the ANOVA results.

Thus, although in vivo proliferation levels were generally consistent with virulence classifications based on larval survival, they were not sufficient on their own to statistically discriminate between the *E. coli* strains. These findings highlight the complexity of host‐pathogen interactions and suggest that bacterial load alone does not necessarily reflect the overall virulence of the strains.

##### 3.3.3.2 Klebsiella Sp

**Figure 10:**
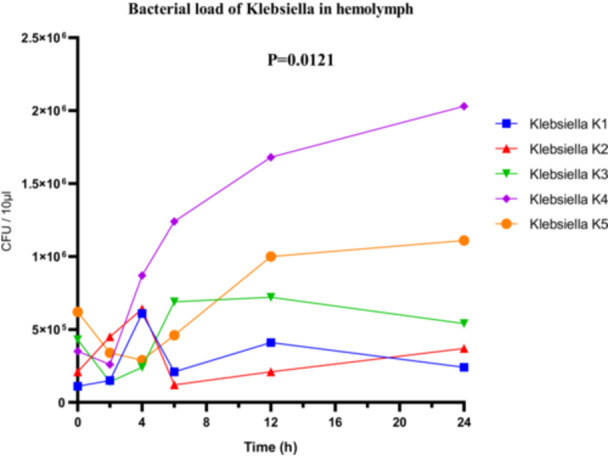
Bacterial load (CFU/10 µL hemolymph) in *R. phoenicis* larvae following infection with Klebsiella strains (K1, K2, K3, K4, and K5). A significant difference was observed between groups (*p* = 0.0121). CFU = colony‐forming units.

In *Klebsiella* sp., a clear variation in bacterial loads was also observed. The highly virulent strain K4 reached a maximum load of 2.1 × 10^6^ CFU/µL (Figure 10), while the low‐virulence strain K5 showed lower proliferation, with 1.1 × 10^6^ CFU/µL. ANOVA revealed a statistically significant difference in bacterial loads among the different strains (*F*_(4, 25)_ = 4.003; *p* = 0.0121; *R* ^2^ = 0.3904). However, both the Brown‐Forsythe test (*F*_(4, 25)_ = 5.272; *p* = 0.0032) and Bartlett's test (corrected statistic = 13.31; *p* = 0.0099) indicated significant heterogeneity of variances, suggesting that interpretation should be approached with caution and that robust post hoc analyses adapted to unequal variances may be warranted (Figure 10).

##### 3.3.3.3 Salmonella Sp

**Figure 11:**
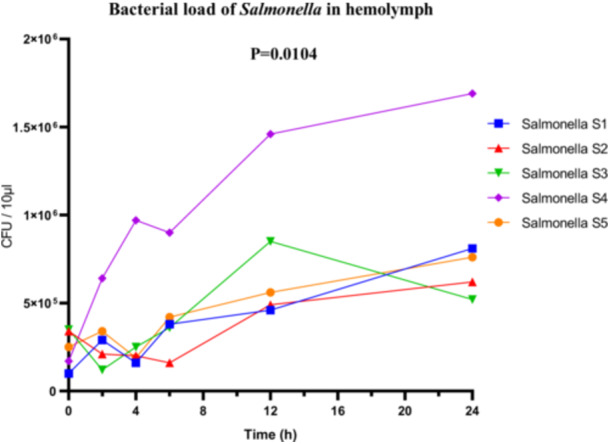
Bacterial load (CFU/10 µL hemolymph) in *R. phoenicis* larvae following infection with Salmonella strains (S1, S2, S3, S4, and S5). A significant difference was observed between groups (*p* = 0.0104). CFU = colony‐forming units.

For the *Salmonella* sp. strains, the highly virulent strain S4 reached a maximum bacterial load of 1.6 × 10^6^ CFU/µL in the hemolymph. Although the bacterial loads of the other strains were not detailed, ANOVA revealed significant overall differences between the strains (*F*_(4, 25)_ = 4.140; *p* = 0.0104; *R* ^2^ = 0.3984). The Brown‐Forsythe test (*F*_(4, 25)_ = 1.724; *p* = 0.1761) and Bartlett's test (corrected statistic = 8.122; *p* = 0.0872) did not detect significant variance heterogeneity, supporting the validity of the statistical conclusions (Figure 11).

##### 3.3.3.4 Enterobacter Sp

**Figure 12:**
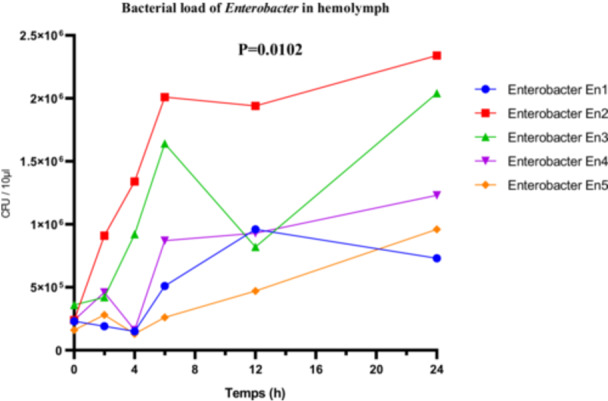
Bacterial load (CFU/10 µL hemolymph) in *R. phoenicis* larvae following infection with Enterobacter strains (En1, En2, En3, En4, and En5). CFU = colony‐forming units.

The *Enterobacter* sp. strains exhibited marked differences in their proliferation within the hemolymph. The highly virulent strain En2 reached a maximum bacterial load of 2.5 × 10^6^ CFU/µL. The moderately virulent strains En1 and En4 showed respective loads of 0.7 × 10^6^ and 1.3 × 10^6^ CFU/µL, supporting the correlation between virulence and multiplication capacity within the host. Statistical analysis using one‐way ANOVA revealed a significant difference among the bacterial loads of the different strains (*F*_(4, 25)_ = 4.157; *p* = 0.0102; *R* ^2^ = 0.3995). The Brown‐Forsythe test (*F*_(4, 25)_ = 1.929; *p* = 0.1368) and Bartlett's test (corrected statistic = 6.402; *p* = 0.1711) did not indicate violations of homogeneity of variance assumptions, thus validating the ANOVA results (Figure 12).

#### 3.3.4 Analysis of Body Melanization Extent in Larvae

The larvae inoculated with various Enterobacteriaceae strains showed varying levels of melanization after experimental infection. Melanization spots were observed in all test groups, with the exception of the control samples and those treated with PBS. In particular, melanization was particularly pronounced in larvae infected with the most virulent strains.

**Figure 13:**
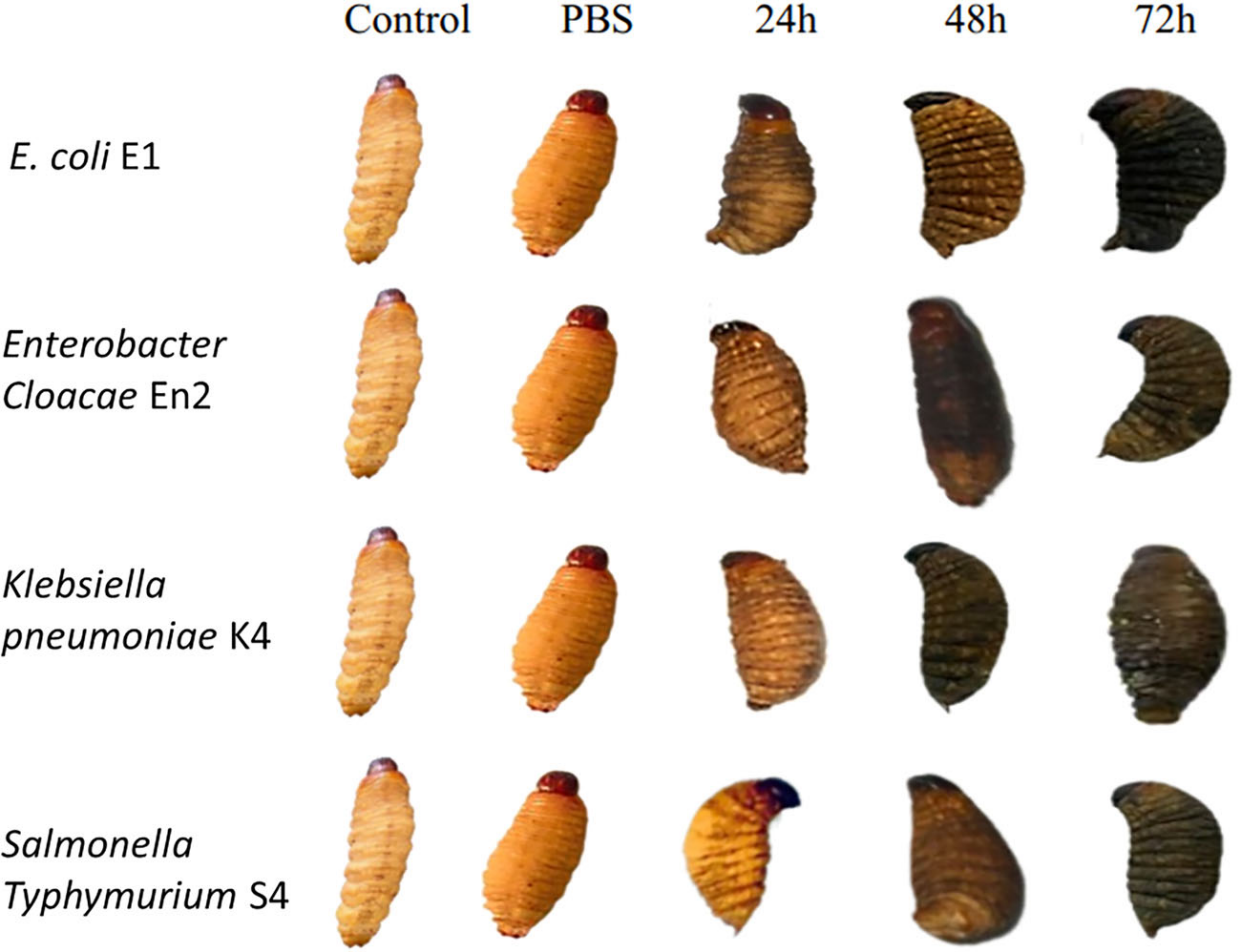
Time‐course analysis of melanization induced by the bacterial strains *S. Typhimurium* (S4), *K. pneumoniae* (K4), *E. cloacae* (En2), and *E. coli* (E1). PBS = phosphate‐buffered saline.

Melanization in *Galleria mellonella* larvae was quantified by analyzing grayscale values, where lower values indicate darker pigmentation and thus more intense melanization. In the control groups (Control and PBS), the mean grayscale values were 186 and 127, respectively, indicating an absence or minimal effect of melanization. Following exposure to various bacterial strains for 24–72 h, a progressive reduction in grayscale values was observed, reflecting activation of the larvae's immune response (Figure 13).

**Figure 14:**
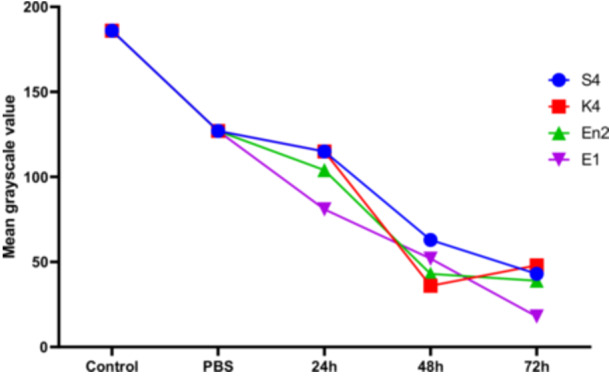
Grayscale intensity curve of larval melanization. The y‐axis represents the level of larval melanization, while the x‐axis indicates the time course of melanization. *S. Typhimurium* (S4), *K. pneumoniae* (K4), *E. cloacae* (En2), and *E. coli* (E1) correspond to the bacterial strains tested in this experiment.

At 24 h, the mean grayscale value was 103.75, indicating the onset of melanization. This response intensified at 48 h (mean: 48.5) and peaked at 72 h with a mean value of 37. At this final time point, marked differences were observed between strains: strain E1 induced the strongest melanization (value: 18), followed by En2 (39), S4 (43), while K4 triggered a slightly weaker response (48). These results indicate that larval melanization is time‐dependent and potentially influenced by the virulence level of each bacterial strain (Figure 14).

### 3.4 Combination of Biosurfactants Secreted by *B. Subtilis* and Amoxicillin

#### 3.4.1 Antibacterial Effects of Biosurfactant

**Figure 15:**
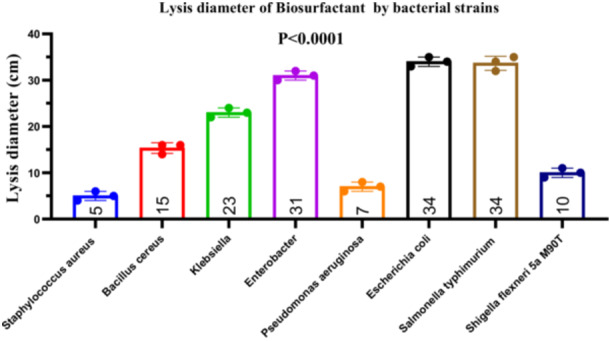
Bacterial growth inhibition diameters of *B. subtilis* biosurfactant extract. The y‐axis represents the diameter of the lysis zones in centimeters. The x‐axis indicates the bacterial strains used in this experiment. Data are presented as mean ± standard deviation (SD). A significant difference was observed between groups (*p* < 0.0001). BS = biosurfactant, cm = centimeter.

To demonstrate the production of biosurfactants with antibacterial activity, *Bacillus subtilis* was subjected to an emulsification test, which yielded a high emulsification index (EI_424_) of 90% after 24 h (data not shown). The crude biosurfactant extract was tested against eight bacterial pathogens, revealing inhibition zones ranging from 2 to 35 mm. The mean inhibition diameters (± standard deviation) were as follows: *S. aureus* (5.0 ± 1.0 mm), *B. cereus* (15.33 ± 1.15 mm), *Klebsiella sp*. (23.0 ± 1.0 mm), *Enterobacter sp*. (31.0 ± 1.0 mm), *P. aeruginosa* (7.0 ± 1.0 mm), *E. coli* (34.0 ± 1.0 mm), *S. typhimurium* (33.67 ± 1.53 mm), and *S. flexneri* (10.0 ± 1.0 mm) (Figure 15). A one‐way ANOVA revealed a statistically significant difference among the groups (*F*_(7, 16)_ = 364.8, *p* < 0.0001, *R* ^2^ = 0.9938), indicating heterogeneous susceptibility of the tested bacteria to the biosurfactant. Post hoc analysis showed significantly greater inhibition of *E. coli* and *S. typhimurium* compared to other pathogens (*p* < 0.05). The Brown–Forsythe test for homogeneity of variances yielded *F*_(7, 16)_ = 0.077, *p* = 0.9989, indicating no significant differences in standard deviations (ns). These results were further confirmed by the non‐significance in Bartlett's test (data not shown), supporting the assumption of equal variances among groups (Figure 15).

#### 3.4.2 Combined Effect of Biosurfactant and Amoxicillin Against Enterobacteria

##### 3.4.2.1 *E. coli* (Strain E1)

**Figure 16:**
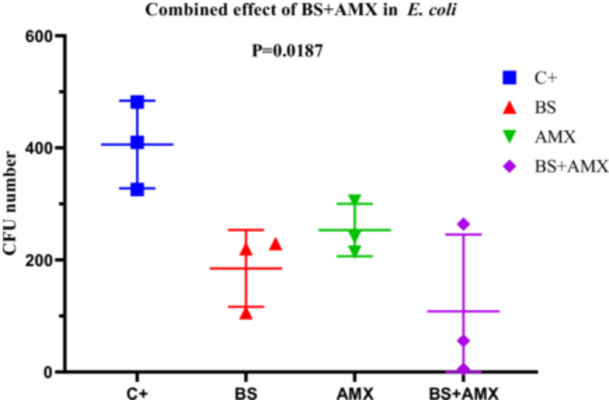
The effect of combination of biosurfactants (BS) secreted by *B. subtilis* and amoxicillin (AMX) against *E. coli* E1. The y‐axis represents the colony‐forming units (CFU). The x‐axis indicates the tested samples, including C+ (positive control), BS (biosurfactant), and BS + AMX (a combination of the biosurfactant and amoxicillin). A significant difference was observed between groups (*p* = 0.0187).

The MIC of amoxicillin against *E. coli* E1 was determined to be 860 µg/mL. Treatment with biosurfactin alone (BS, 40 µL) or amoxicillin alone (AMX, 250 µg/mL) moderately reduced bacterial colony formation (CFUs) over time. However, the combination of both agents (BS + AMX) proved significantly more effective. At 4 h, the CFU count dropped from 482 (positive control, C+) to just 5 with the combined treatment. ANOVA analysis revealed a significant difference between groups (*p* = 0.0187; *F*_(3, 8)_ = 6.047; *R* ^2^ = 0.6940), although the Brown‐Forsythe (*p* = 0.7736) and Bartlett tests did not show significant variance differences among groups (*p* > 0.05). These results suggest that the combined treatment exhibits notable synergy compared to individual treatments (Figure 16).

##### 3.4.2.2 Salmonella Typhimurium (Strain S4)

**Figure 17:**
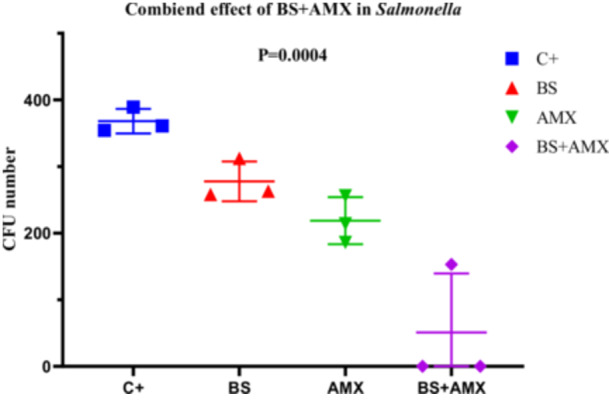
The effect of combination of biosurfactants (BS) secreted by *B. subtilis* and amoxicillin (AMX) against *S. typhymurium* S4. The y‐axis represents the colony‐forming units (CFU). The x‐axis indicates the tested samples, including C+ (positive control), BS (biosurfactant), and BS + AMX (a combination of the biosurfactant and amoxicillin). A significant difference was observed between groups (*p* = 0.0004).

The amoxicillin MIC for *Salmonella Typhimurium* S4 was determined to be 640 µg/mL. The combined treatment (BS + AMX) led to complete inhibition of bacterial growth as early as the 2nd hour (CFU = 0), whereas individual treatments showed only partial effectiveness. ANOVA analysis confirmed a highly significant difference between the groups (*p* = 0.0004; *F*_(3, 8)_ = 20.81). The strong reduction in bacterial load with the combined treatment highlights a remarkable synergistic effect, particularly in this strain. None of the variability tests (Bartlett, Brown‐Forsythe) revealed significant differences between standard deviations, validating the homogeneity of the data (Figure 17).

##### 3.4.2.3 *Klebsiella Pneumoniae* (Strain K4)

**Figure 18:**
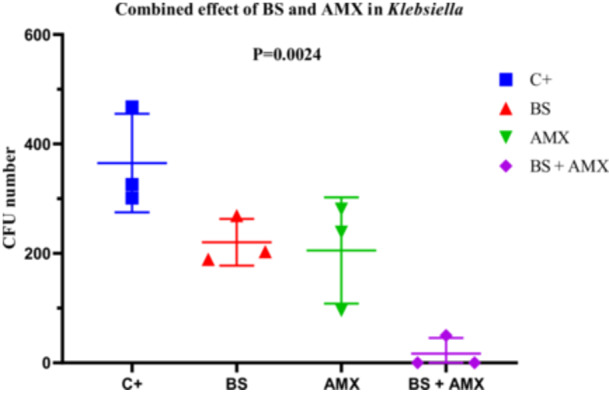
The effect of combination of biosurfactants (BS) secreted by *B. subtilis* and amoxicillin (AMX) against *K. pneumoniae* K4. The y‐axis represents the colony‐forming units (CFU). The x‐axis indicates the tested samples, including C+ (positive control), BS (biosurfactant), and BS + AMX (a combination of the biosurfactant and amoxicillin). A significant difference was observed between groups (*p* = 0.0024).

The MIC of amoxicillin against *K. pneumoniae* K4 was 580 µg/mL. While amoxicillin alone led to a gradual reduction in CFU counts (from 281 to 96), the combination with the biosurfactant resulted in complete inhibition as early as the 2nd hour, which was maintained at the 4‐h mark (CFU = 0). ANOVA revealed a statistically significant difference (*p* = 0.0024; *F*_(3, 8)_ = 12.14), confirming the enhanced efficacy of the combined treatment. No significant differences in variance were observed according to Bartlett's and Brown‐Forsythe tests. These findings support the potential of a combined therapeutic approach for controlling infections caused by multidrug‐resistant *K. pneumonia* (Figure 18).

##### 3.4.2.4 *E. Cloacae* (Strain En2)

**Figure 19:**
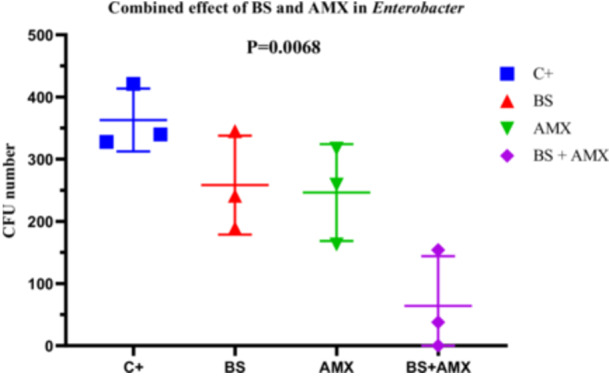
The effect of combination of biosurfactants (BS) secreted by *B. subtilis* and amoxicillin (AMX) against *E. cloacae* En2. The y‐axis represents the colony‐forming units (CFU). The x‐axis indicates the tested samples, including C+ (positive control), BS (biosurfactant), and BS + AMX (a combination of the biosurfactant and amoxicillin). A significant difference was observed between groups (*p* = 0.0068).

For *E. cloacae* En2, the MIC of amoxicillin was measured at 420 µg/mL. The BS + AMX combination led to complete inhibition of bacterial growth at 4 h (CFU = 0), in contrast to the individual treatments, which showed intermediate efficacy. ANOVA revealed a significant difference between treatments (*p* = 0.0068; *F*_(3, 8)_ = 8.669; *R* ^2^ = 0.7648). Variability tests did not show significant differences in standard deviations (Brown‐Forsythe *p* = 0.9449). These results indicate a strong synergy between biosurfactin and amoxicillin in inhibiting *E. cloacae*, particularly during the early hours of treatment (Figure 19).

#### 3.4.3 In Vivo Survival Test of *R. Phoenicis* Larvae in the Presence of the Two Bioactive Agents

##### 3.4.3.1 In Vivo Survival Test of *R. Phoenicis* Against *E. coli* E1 in the Presence of Bioactive Compounds

The virulence of *E. coli* strain E1 was assessed by monitoring larval survival over a 72‐h period. In the untreated group, survival dropped sharply from 100% at 0 h to 30% at 24 h, then to 20% at 48 h, and ultimately reached 0% at 72 h. When treated with amoxicillin alone, survival remained modest, with 50% at 24 h and 30% persisting through to 72 h. The biosurfactant alone showed a slight improvement, with survival at 60% at 24 h, 40% at 48 h, but declined to 0% at 72 h. However, the combination of amoxicillin and biosurfactant yielded the most protective effect, maintaining 80% survival at 24 h, 70% at 48 h, and 50% at 72 h.

**Figure 20:**
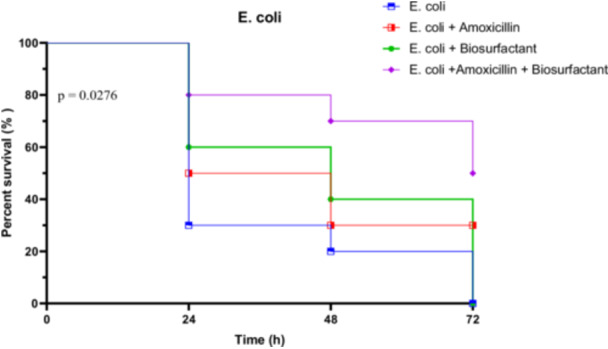
In vivo survival of *R. phoenicis* larvae over 72 h post‐infection with *E. coli* E1, with or without amoxicillin (AMX) and biosurfactant (BS) treatment. A significant difference was observed between groups (*p* = 0.0276). AMX = amoxicillin, BS = biosurfactant, h = hours.

Statistical analyses confirmed significant differences between treatment groups. The Log‐rank (Mantel‐Cox) test showed a Chi‐square of 9.134 (*p* = 0.0276), indicating significantly different survival curves. The trend test was also significant (*p* = 0.0104), while the Gehan‐Breslow‐Wilcoxon test approached significance (*p* = 0.0537). These results highlight the superior efficacy of the combined treatment in mitigating *E. coli* E1 virulence (Figure 20).

##### 3.4.3.2 In Vivo Survival Test of *R. Phoenicis* Against *K. Pneumoniae* K4 in the Presence of Bioactive Compounds

Larvae infected with *K. pneumoniae* strain K4 exhibited a moderate decline in survival under all conditions, with untreated larvae showing 80% survival at 24 h, dropping to 20% at 72 h. Treatment with amoxicillin alone did not significantly improve outcomes, with survival falling from 80% at 24 h to just 10% at 72 h. Biosurfactant treatment yielded similar trends, starting at 50% and ending at 20%. The amoxicillin + biosurfactant combination maintained a relatively higher protection, with 60% at 24 h and 50% persisting through to 72 h.

**Figure 21:**
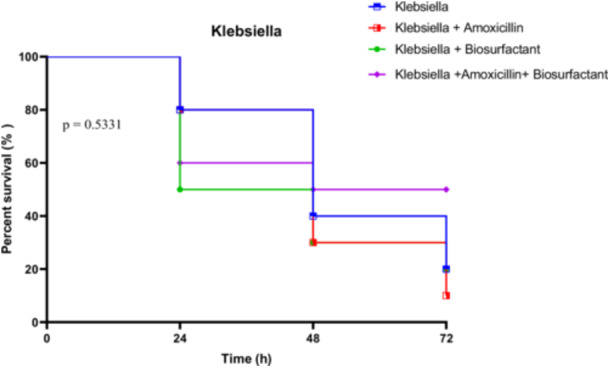
In vivo survival of *R. phoenicis* larvae over 72 h post‐infection with Klebsiella K4, with or without amoxicillin (AMX) and biosurfactant (BS) treatment. No significant difference was observed between groups (*p* = 0.5331). AMX = amoxicillin, BS = biosurfactant, h = hours.

However, statistical analysis indicated no significant differences among the treatment groups. The Log‐rank test gave a Chi‐square of 2.194 (*p* = 0.5331), the trend test was also non‐significant (*p* = 0.4849), and the Gehan‐Breslow‐Wilcoxon test confirmed this absence of significance (*p* = 0.7178). These results suggest a more limited impact of the treatments, possibly due to inherent resistance traits in *K. pneumoniae* K4 (Figure 21).

##### 3.4.3.3 In Vivo Survival Test of *R. Phoenicis* Against *Salmonella Typhimurium* S4 in the Presence of Bioactive Compounds

Survival of larvae infected with *S. typhimurium* S4 followed a similar pattern of improvement under treatment, particularly with the combined therapy. Untreated larvae showed rapid decline: 60% at 24 h, 40% at 48 h, and 0% at 72 h. Amoxicillin alone provided moderate protection (70% at 24 h, 50% at 48 h, and 20% at 72 h), while the biosurfactant alone was less effective (60%, 40%, and 10%, respectively). The combination therapy proved superior, maintaining 80% survival at 24 h, and notably 70% at 72 h.

**Figure 22:**
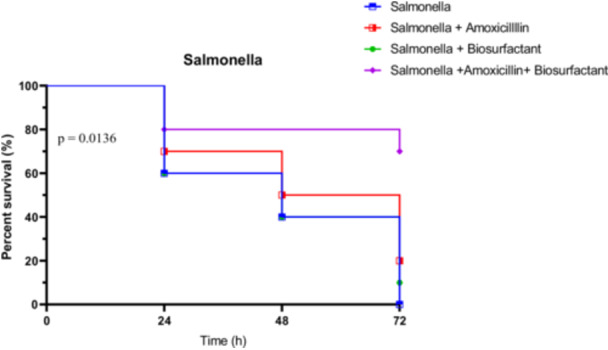
In vivo survival of *R. phoenicis* larvae over 72 h post‐infection with Salmonella S4, with or without amoxicillin (AMX) and biosurfactant (BS) treatment. A significant difference was observed between groups (*p* = 0.0136). AMX = amoxicillin, BS = biosurfactant, h = hours.

Statistical evaluation supported these findings. The Log‐rank test was significant (*χ* ^2^ = 10.68, *p* = 0.0136), and the trend test showed a strong trend (*p* = 0.0069). The Gehan‐Breslow‐Wilcoxon test did not reach significance (*p* = 0.0574), but was close. These data highlight the effectiveness of the combined treatment in reducing *Salmonella* S4 virulence in vivo (Figure 22).

##### 3.4.3.4 In Vivo Survival Test of *R. Phoenicis* Against *E. Cloacae* En2 in the Presence of Bioactive Compounds

**Figure 23:**
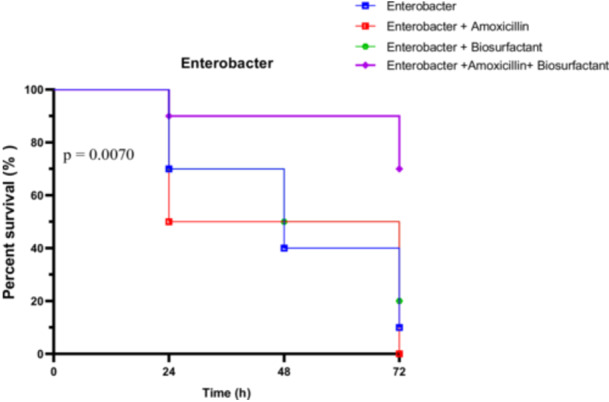
In vivo survival of *R. phoenicis* larvae over 72 h post‐infection with Enterobacter En2, with or without amoxicillin (AMX) and biosurfactant (BS) treatment. A significant difference was observed between groups (*p* = 0.0070). AMX = amoxicillin, BS = biosurfactant, h = hours.

*E. cloacae* strain En2‐infected larvae showed notable benefit from the combined therapy. Untreated larvae had survival rates of 70% at 24 h, dropping to 10% at 72 h. Amoxicillin alone resulted in a sharper decline (50% at 24 h, 0% at 72 h), while the biosurfactant alone maintained 70% at 24 h and 20% at 72 h. The combined treatment offered the highest protection, starting at 90% and ending with 70% survival at 72 h (Figure 23).

All statistical tests confirmed significant survival differences among groups. The Log‐rank test yielded a Chi‐square of 12.10 (*p* = 0.0070), the trend test showed a clear directional effect (*p* = 0.0023), and the Gehan‐Breslow‐Wilcoxon test was also significant (*p* = 0.0170). These results emphasize the potent efficacy of the amoxicillin + biosurfactant combination against *E. cloacae* En2 in vivo.

## 4 Discussion

In most studies, scientific results are often presented in isolation, without contextualizing the overall experimental framework. In contrast, the present study distinguishes itself by situating the investigation within a comprehensive experimental context and by employing a wide range of microbiological and molecular approaches to assess the pathogenicity of Enterobacteriaceae, including *E. coli*, *Klebsiella spp*., *Salmonella spp*., and *Enterobacter spp*. Classical microbiological assays—such as hemolysis, resistance to gastric acidity, biosurfactant production, biofilm formation, and swarming motility—were employed to evaluate the virulence profiles of clinical isolates. The enteric bacteria analyzed in this study encompass strict pathogens (e.g., *Salmonella* spp.) (Kylla et al. 2019), opportunistic or conditionally pathogenic species like *Klebsiella* (Wang et al. 2020), and commensal strains like *E. coli*, which can become pathogenic under certain circumstances (Pakbin et al. 2021). This stratification highlights the diverse pathogenic potential of Enterobacteriaceae across clinical contexts.

Animal models have historically driven advances in infectious disease research, providing crucial insights into host–pathogen interactions (Goh et al. 2017; Calum et al. 2022; Tsai et al. 2016; Prakoso et al. 2022). Mammalian models, despite offering physiological relevance, pose significant logistical and ethical challenges. In recent years, insect larvae—particularly *Galleria mellonella*—have gained popularity as alternative infection models due to their lower cost, ease of handling, and ethical acceptability (Graves 2012; Murayama et al. 1986; Kaito et al. 2020). By drafting this paper, *G. mellonella* larvae up to date commonly used to study bacterial pathogenicity (Asai et al. 2023; Tsai et al. 2016; Prakoso et al. 2022).

In this context, we introduce *R. phoenicis* larvae as a novel invertebrate model for studying the virulence of Enterobacteriaceae. This species presents several advantages: it is endemic to sub‐Saharan Africa, widely available, suitable for storage at room temperature, and tolerates incubation at 37°C—a critical temperature for the expression of many human bacterial virulence factors. Additionally, these larvae are commonly used as a food source due to their high nutritional value (Okouakoua et al. 2024; Ayensu et al. 2020; Niod et al. 2024; Ayensu et al. 2019; Chamoun et al. 2023).

Our results show that larval melanization occurs within 24 h post‐infection, indicating high virulence of the tested isolates, particularly *Salmonella*. Epidemiologically, stool samples revealed a higher prevalence of infection in children aged 0–5 years, a group especially vulnerable to diarrheal diseases (Kombat et al. 2024; Gessesse and Tarekegn 2022). Co‐infections involving two or more bacterial species were also detected, consistent with previous observations in diarrheal pathologies (Lee et al. 2023; Vergadi et al. 2021). While *Klebsiella*, *Enterobacter*, and *E. coli* were present in smaller proportions, *Salmonella* was predominant, corroborating its role as a leading causative agent of enteric infections (Wang et al. 2022; Talukder et al. 2023).

Molecular analysis revealed the presence of genes encoding T3SSs, which are key virulence determinants in Gram‐negative pathogens (Dos Santos et al. 2020; Kayath et al. 2010). These systems facilitate the direct injection of effector proteins into host cells, enhancing bacterial invasion and immune evasion (Kato et al. 2018). The observed melanization in *R. phoenicis* larvae may be attributed to the expression of such virulence mechanisms (Bertani and Ruiz 2018; Nakano 2012; Kinouani Kinavouidi et al. 2020). Moreover, most strains exhibited significant resistance to acidic pH, with survival rates ranging from 10% to 90%, supporting their ability to persist in hostile environments like the gastrointestinal tract (Kinavouidi et al. 2022; Liu et al. 2022; Kosilova et al. 2020). These results underscore significant variations in the biological characteristics of enterobacterial strains, providing valuable insights into their virulence and persistence.

Experimental infections demonstrated a clear dose‐response relationship. Injections of ≥ 10^5^ CFU/µL resulted in complete larval mortality within 24 h, whereas lower doses (10^4^ CFU/µL) produced negligible lethality. These findings align with previous studies using *G. mellonella*, where bacterial concentration significantly influenced mortality rates (Ayensu et al. 2019; Chamoun et al. 2023; Chen and Keddie 2021). Statistical analysis confirmed significant differences in virulence among strains, as reflected by variations in both melanization and hemolymph bacterial load. These results highlight the utility of *R. phoenicis* as a sensitive model for differentiating strain virulence (da Silva et al. 2024; Banfi et al. 2024; Younes et al. 2020; Verster et al. 2023).

Importantly, the combination of a *B. subtilis*‐derived biosurfactant and amoxicillin demonstrated synergistic antibacterial activity. These findings align with and extend previous studies demonstrating synergistic interactions between biosurfactants and antibiotics, particularly against biofilm‐forming or antibiotic‐resistant bacteria (Juma et al. 2020; Joshi‐Navare and Prabhune 2013b; Lin et al. 2021; Douafer et al. 2021). This study presents a distinctive aspect in that it employs a biosurfactant isolated from *B. subtilis*, typically identified as surfactin (Okouakoua et al. 2024; Li et al. 2023). Extensive research has been conducted on the synergistic effects of glycolipid and lipopeptide biosurfactants in combination with various antibiotics, particularly against bacterial biofilms and antibiotic‐resistant strains (Li et al. 2023; Amirinejad et al. 2022; Rivardo et al. 2011; Puyol McKenna et al. 2024). However, only a limited number of studies have investigated such combinations in animal models, as exemplified by the current work.

We propose that our in vivo model offers a promising platform for rapid, effective, and cost‐efficient experimentation. Despite some unanswered questions, the model remains straightforward and economically viable. In natural conditions, larvae exhibit high survival rates, likely due to the protective function of their integumentary barrier, which resists invasion by pathogenic bacteria. Microinjections were conducted at ambient temperature, demonstrating that strict aseptic conditions are not essential for the procedure. Moreover, the larvae are widely available in the Republic of the Congo, ensuring year‐round accessibility. However, a notable limitation is the reliance on palm tree felling to obtain *R. phoenicis* larvae, raising sustainability concerns.

The observed reduction in larval survival following infection with highly virulent strains of *E. coli* (EPEC E2), *K. pneumoniae* (K4), *S. Typhimurium* (S4), and *E. cloacae* (En2) at a bacterial concentration of 10⁵ CFU/µL demonstrates a robust pathogenic effect in *R. phoenicis* larvae. The magnitude of mortality, coupled with hemolymph bacterial loads reaching 2.5 × 10^5^ CFU/µL, indicates that these larvae are not only permissive to infection but are also capable of mounting a measurable biological response—thus validating their use as a sensitive in vivo infection model.

Biologically, the clear distinction between highly virulent and less virulent strains mirrors findings from established invertebrate models such as *G. mellonella* (Kay et al. 2019) and *Tenebrio molitor*, where survival and bacterial load are used as reliable correlations of pathogen virulence. In *G. mellonella*, similar CFU ranges (10^4^–10^6^ CFU/larva) have been shown to induce significant mortality when injected with comparable Gram‐negative pathogens, reinforcing the biological relevance of outcome measures.

The enhanced efficacy of the amoxicillin + biosurfactant combination, which sustained larval survival at 70%–80% over 72 h, is also biologically meaningful. This level of therapeutic effect not only exceeds that of amoxicillin monotherapy but is consistent with studies using *G. mellonella*, where combination therapies have demonstrated improved survival outcomes and reduced microbial burden. Such findings support the idea that biosurfactants can potentiate antibiotic action, possibly through enhanced membrane permeability and inhibition of resistance mechanisms.

The observed effects in *R. phoenicis* larvae are both statistically and biologically significant. The results are in alignment with findings from other invertebrate infection models, reinforcing the validity of *R. phoenicis* as a novel and effective model for studying host–pathogen interactions and evaluating antimicrobial treatments (Siddiqui et al. 2024).

Building on these promising findings, several future research directions are warranted. Firstly, evaluating the effectiveness of other antibiotic classes—such as aminoglycosides, fluoroquinolones, or carbapenems—in combination with biosurfactants could provide broader insight into potential synergistic interactions. Secondly, a deeper exploration of the *R. phoenicis* immune response at the molecular level is essential. Additionally, histopathological studies of larval tissues post‐infection and treatment could offer valuable insights into the physiological impact and recovery dynamics. Finally, scaling up this model for use in medium‐throughput screening platforms, especially in resource‐limited settings, could contribute significantly to antimicrobial drug development tailored for sub‐Saharan contexts.

## 5 Conclusion

In conclusion, *R. phoenicis* larvae prove to be an effective model for studying host‐pathogen interactions and may provide a useful alternative to more costly and complex models. The results also highlight the importance of the amoxicillin‐biosurfactant combination in enhancing therapeutic efficacy and manage infections caused by enterobacteria.

## Author Contributions

**Sergy Patrick Junior Bissoko:** conceptualization, methodology, resources, writing – original draft, investigation. **Christian Aimé Kayath:** conceptualization, methodology, software, data curation, supervision, project administration, writing – review & editing, validation. **Saturnin Nicaise Mokemiabeka:** data curation, supervision, resources, formal analysis. **Frédéric Yannick Okouakoua:** data curation, supervision, resources, formal analysis, visualization. **David Charles Roland Moukala:** data curation, formal analysis, validation, writing – original draft. **Duchel Jeanedvi Kinouani Kinavouidi:** data curation, software, formal analysis.

## Ethics Statement

The authors have nothing to report.

## Conflicts of Interest

The authors declare no conflicts of interest.

## Data Availability

Data sharing not applicable to this article as no datasets were generated or analysed during the current study.
